# Protein-Restricted Diets for Ameliorating Motor Fluctuations in Parkinson's Disease

**DOI:** 10.3389/fnagi.2017.00206

**Published:** 2017-06-28

**Authors:** Luxi Wang, Nian Xiong, Jinsha Huang, Shiyi Guo, Ling Liu, Chao Han, Guoxin Zhang, Haiyang Jiang, Kai Ma, Yun Xia, Xiaoyun Xu, Jie Li, Jing Y. Liu, Tao Wang

**Affiliations:** ^1^Department of Neurology, Union Hospital, Tongji Medical College, Huazhong University of Science and TechnologyWuhan, China; ^2^Key Laboratory of Molecular Biophysics of the Ministry of Education, College of Life Science and Technology and Center for Human Genome Research, Huazhong University of Science and TechnologyWuhan, China

**Keywords:** Parkinson's disease, diet therapy, protein, fluctuation, levodopa

## Abstract

Levodopa is considered to be the most effective symptomatic drug for Parkinson's disease (PD). As the disease progresses, however, the patients are likely to experience a reduced response to levodopa and develop motor fluctuations (i.e., end-of-dose wearing off and unpredictable “on-off”). Protein-rich diets and elevated plasma concentrations of large neutral amino acids have been proved to impair the therapeutic effect of levodopa by reducing its absorption and influx into the brain. On the other hand, the protein-restricted diets including low-protein diet (LPD), protein-redistribution diet (PRD) and PRD with use of low-protein products can all improve the efficacy of levodopa in patients with motor fluctuations. However, it should be noted that protein-restricted diets may also contribute to several side effects, including dyskinesia, weight loss, and malnutrition (e.g., protein and calcium insufficiency). Together, protein-restricted diets are an effective approach to improve motor fluctuations in PD patients, while long-term adherence to these diets requires monitoring for side effects.

## Introduction

Parkinson's disease (PD) is the second common neurodegenerative disease characterized by motor and non-motor symptoms (Kalia and Lang, 2015; Ascherio and Schwarzschild, 2016). Motor symptoms of the disease include bradykinesia, muscular rigidity, rest tremor, as well as postural, and gait impairment (Kalia and Lang, 2015). At present, levodopa (LD) is the most effective treatment for PD patients, and is commonly regarded as the “gold standard” of PD therapy (Connolly and Lang, 2014; LeWitt, 2015). However, with prolonged treatment, the patients are likely to show a reduced response to levodopa, and develop motor complications including motor fluctuations and dyskinesia (Jankovic and Stacy, 2007; Kalia and Lang, 2015). Usually, the patients with motor fluctuations initially experience progressively shorter response to each dose of levodopa (end-of dose wearing-off), and subsequently show abrupt unpredictable shifts between “on” and “off” states which are not related to drug administration (“on-off” phenomenon; Adler, 2002). After 5 years of levodopa treatment, motor fluctuations are encountered in nearly 40% of the patients (Jankovic, 2005). Fluctuating motor performance is the major source of disability in PD patients, and significantly impairs their quality of life (QOL; Chapuis et al., 2005; Espay, 2010; Rascol et al., 2011).

Though levodopa provides the greatest symptomatic benefit for PD, there is an ongoing debate whether the initiation of levodopa therapy should be delayed to postpone the onset of levodopa-associated motor complications (Cilia et al., 2014; Connolly and Lang, 2014; Gray et al., 2014; Lang and Marras, 2014; Kalia and Lang, 2015; Yahalom et al., 2016). For instance, a retrospective analysis on 170 PD patients found early levodopa administration was related with an earlier onset of motor fluctuations from onset of PD, suggesting the need to delay the initiation of levodopa therapy in parkinsonians (Yahalom et al., 2016). On the other hand, a large open-label randomized trial in newly-diagnosed PD patients showed small but persistent benefit from initial treatment with levodopa, compared with initial treatment with a dopamine agonist or a MAO-B inhibitor (i.e., levodopa-sparing therapy; Gray et al., 2014). Despite controversy, many clinicians will continue to use an initial levodopa-sparing therapy in most patients with early onset of PD, to delay the emergence of motor fluctuations and dyskinesias (Lang and Marras, 2014; Kalia and Lang, 2015).

Therefore, maximizing the therapeutic efficiency of levodopa and controlling motor fluctuations, is still an important but challenging goal in the treatment of PD. Due to a significant interaction between dietary proteins and patients' response to levodopa, a large number of studies have investigated the clinical effects of protein-restricted diets in fluctuating parkinsonians since 1970s. In this review, we discuss the changes in the pharmacokinetics of levodopa related to dietary proteins, and summarize the recent and historical studies that investigated the effects of high-protein meals and protein-restricted diets in parkinsonian patients or animal models. References were identified by searches of PubMed through March 2017 for English-language primary studies and systematic reviews, using the search terms “Parkinson's disease,” “levodopa,” “motor fluctuations,” “protein intake,” “protein diet,” “protein restriction,” “large neutral amino acid,” and their synonyms.

## Dietary influences on levodopa pharmacokinetics

Levodopa is absorbed by a saturable facilitated large neutral amino acid (LNAA) transport system, and most of the absorption takes place in small intestine (Contin and Martinelli, 2010). As levodopa is rapidly metabolized to dopamine in gastrointestinal tract by amino acid decarboxylase (AADC), only 30% of orally-administered levodopa reached the systemic circulation when given alone (Khor and Hsu, 2007; Contin and Martinelli, 2010). Therefore, levodopa is commonly co-administered with AADC inhibitors such as carbidopa (CD) and benserazide in the treatment of PD (Bianchine et al., 1972; Khor and Hsu, 2007). In healthy subjects, fasting levodopa absorption from immediate-release (IR) CD-LD tablets is very rapid, with 90% of the oral dose taken up in the first hour, and the absorption was almost complete in 2 h (Khor and Hsu, 2007; LeWitt, 2015).

Levodopa pharmacokinetics show high inter- and intrasubject variability and can be significantly influenced by food intake (Contin and Martinelli, 2010; Ramirez-Zamora and Molho, 2014; LeWitt, 2015). When administered half an hour after a meal, levodopa exhibits an extended t_max_ by 2- to 3-fold, a decreased plasma C_max_ by 30%, and a smaller AUC by 15% (Baruzzi et al., 1987). On one hand, meals slow gastric emptying and delay the transfer of levodopa to the proximal small intestine, where most of its absorption takes place (Mearrick et al., 1974; Nutt et al., 1984; Baruzzi et al., 1987; LeWitt, 2015). A stimulation of gastric motility by metoclopramide can nearly triple C_max_ of levodopa, and lead to a 1.5 to 2-fold increase in its bioavailability, which indicates the rate of gastric emptying has considerable influences on the absorption of levodopa (Mearrick et al., 1974). On the other hand, dietary LNAAs may compete with levodopa for the saturable LNAA transport system in the gut, and thus impair the bioavailability of the drug (Contin and Martinelli, 2010). For instance, the LNAA L-leucine has been reported to decrease the intestinal absorption of levodopa by ~50% in healthy subjects (Lennernas et al., 1993).

Levodopa crosses the blood brain barrier (BBB) via the same saturable LNAA system as in the intestine (Contin and Martinelli, 2010), and also competes with LNAAs for transport from plasma to the brain (Wade and Katzman, 1975; Leenders et al., 1986b; Stout et al., 1998). 2-aminobicyclo(2,2,1) heptane-2-carboxylic acid (BCH), a model substrate of leucine, was found to inhibit the transport of levodopa into the brain by 46–52% in rats (Wade and Katzman, 1975). By positron emission tomography (PET), Leenders and coworkers found the brain uptake of 6-[18F]fluoro-L-dopa (FDOPA) was approximately three times less when loading with amino acids, compared with the uptake in a fasted state (Leenders et al., 1986b). Moreover, BBB influx rate constant of levodopa was found to negatively correlate with plasma LNAA concentrations (*r*^2^ = 0.51) (Stout et al., 1998). After protein ingestion, AUC of levodopa tends to increase greatly while C_max_ and t_max_ of levodopa exhibit no significant changes (Robertson et al., 1991; Simon et al., 2004). It indicates that even though the absorption process of levodopa is not significantly affected by protein load, the fraction of levodopa transported into the brain seems to decrease due to changes in plasma LNAA levels. Together, it is suggested that compared with the similar competition in intestinal mucosa, the competition between levodopa and LNAAs at BBB is the major contributor to variability in levodopa pharmacokinetics related to dietary proteins.

## Influences of dietary proteins on therapeutic effect of levodopa

In patients with early PD, there is no clear-cut concentration-effect relationship of levodopa, which is sometimes referred to as the honeymoon period (Contin and Martinelli, 2010; LeWitt, 2015). However, as PD progresses, while the pharmacokinetics parameters of levodopa keep unchanged, latency to motor response and duration of effect are both markedly shortened during levodopa treatment (Contin et al., 1996). The motor function of patients in advanced stages becomes closely connected with the rapid rise and fall in levodopa concentrations after each dose, which is manifested as motor fluctuations (Contin and Martinelli, 2010). There is also a decrease in striatal storage capacity of levodopa in parkinsonian patients (Leenders et al., 1986a). The patients with fluctuations, in particular, show a greater decrease in central levodopa storage, which may explain why the inconsistency of levodopa entering the brain is the most common problem in most motor fluctuations (Leenders et al., 1986a; LeWitt, 2015).

Back in the 1970s and 1980s, a considerable amount of literature reported that a daily protein intake of about 1.6 g per kilogram of body weight, or a single load of 0.4 g protein per kilogram, was likely to raise plasma LNAA levels and diminish the response to levodopa in PD patients with motor fluctuations (Mena and Cotzias, 1975; Nutt et al., 1984; Juncos et al., 1987; Pincus and Barry, 1987c; Carter et al., 1989). These findings provide the earliest evidence for the need of a protein-restricted diet in fluctuating parkinsonians. However, all these trials were non-double-blinded and on small samples (*n* = 5 to *n* = 9). By analyzing epidemiologic data from 1952 to 1958 in 17 countries, Coughlin *et al*. found the age-adjusted PD mortality rates were positively correlated with total protein intake per capita and meat consumption, implying an excess of protein intake may play a role in the progression of PD (Coughlin et al., 1992). However, this correlation does not clarify any association between dietary proteins and clinical effect of levodopa. With European Prospective Investigation into Cancer and Nutrition (EPIC) questionnaire, an observational study on 45 Italian parkinsonians found the patients with moderate or severe symptoms tended to have higher daily consumption of protein than those with mild symptoms (Marczewska et al., 2006). The study implies an inverse correlation between protein intake and efficacy of antiparkinsonian treatment, but it has not revealed how dietary proteins influence patients' response to the drugs through further investigation.

Two case reports in 2008 and 2010, respectively, have revealed another interesting finding that protein ingestion by enteral nutrition is also likely to impair the effect of levodopa therapy in parkinsonians (Cooper et al., 2008; Bonnici et al., 2010). Additionally, another case report in 2014 suggested high-protein diets may contribute to more severe consequences other than impaired motor function, such as a coma resulting from a severe “off” state (Arulanantham et al., 2014). However, as these findings are reported in a limited number of cases, they need to be validated by additional studies of larger numbers of patients.

There were no studies exploring the onset, prevalence and manifestation pattern of the protein interaction with levodopa (PIL) in PD patients, until a large study in 2016 provided a deeper insight into the issue (Virmani et al., 2016). In the study, a patient was considered to have PIL if he or she had any of the four following conditions after high-protein meals: longer latency to levodopa response, reduced response or duration of response, dose failures, or earlier “off” with a previously effective dose. By an analysis of clinical records in 1037 PD patients, the study found the prevalence of PIL in patients with motor fluctuations was 52/421 (12.4%), indicating PIL is not a common problem in PD. However, the patients with PIL showed more severe “off” states with more frequent dose failures than other patients (Virmani et al., 2016), showing a correlation between PIL and increased severity of motor fluctuations. The study also found the mean onset of PIL was 12.9 years after the onset of motor symptoms or 8 years after the initiation of levodopa therapy. Moreover, the patients with earlier disease onset or with familial history of PD were more likely to develop PIL (Virmani et al., 2016). With a large sample of parkinsonian patients and well-designed subgroup analysis, the study provides a clearer understanding of the interactions between dietary protein and levodopa efficacy. Furthermore, by investigating PIL onset and identifying patients with a higher risk of PIL, the study also helps to predict when and for whom a protein-restricted diet is most likely to be effective in ameliorating motor fluctuations. However, there are also several limitations to consider when discussing the study. Firstly, it is a retrospective research where clinical records of the subjects may be incomplete or biased. Besides, the prevalence of PIL may be under- or overestimated, as the clinical notes are actually based on subjective reports from outpatients. The authors also have not clarified whether the PIL observed in the study is due to elevated levels of plasma LNAAs or a delayed gastric emptying.

Concerning how high-protein diet affects the therapeutic effect of levodopa, existing evidence is mixed (Eriksson et al., 1984; Nutt et al., 1989; Karstaedt and Pincus, 1993). A study on 12 patients with predictable fluctuations revealed that administration of controlled-release LD/CD (Sinemet CR) with a meal resulted in less fluctuations in levodopa concentrations, but administration in the fasting condition led to higher plasma concentrations of levodopa, which seems to help the patients show better motor response (Roos et al., 1993). However, other studies indicate the fluctuations in plasma levodopa levels play the most important role in motor fluctuations related to high-protein meals. A study on 11 fluctuating patients reported that 90% of the variations in levodopa influx into the brain was due to fluctuations in levodopa concentrations, with the rest 10% due to fluctuations of plasma LNAAs (Nutt et al., 1989). A previous study on 5 patients with marked “on-off” symptoms has shown a similar result (Eriksson et al., 1984). These studies, however, neglected the possibility that dietary LNAAs can also influence plasma levodopa levels, and thus contribute to an impaired motor response indirectly. In 1997, Nutt and coworkers performed another study on 11 fluctuating patients with stage III/V PD. By using constant-rate infusions of levodopa, the study observed a negative correlation between motor function and plasma LNAA levels, and the fluctuating motor response was not related with the minor fluctuations in plasma levodopa or its metabolite 3-O-rnethyldopa (3-OMD) (Nutt et al., 1997). Furthermore, the effect of dietary LNAAs on levodopa efficacy has been furtherly confirmed by another two studies. In animal models, parkinsonian rats have shown a decreased response to levodopa after ingestions of high-concentration LNAAs (Mizuta and Kuno, 1993). Similarly, in two patients with advanced PD, oral administration of the LNAA phenylalanine significantly shortened the duration of motor response during constant infusions of levodopa (Woodward et al., 1993). Therefore, it can be concluded that the fluctuating response to levodopa is at least partially caused by the elevations in LNAA concentrations after high-protein meals. Furthermore, a study in rats has suggested that LNAAs in a protein-rich diet may affect the efficacy of levodopa via reversing the levodopa-induced increase in striatal dopamine by 78% (Brannan et al., 1991). Together, existing studies have provided considerable evidence for the impact of dietary proteins on therapeutic effect of levodopa, and strongly indicate the need of protein-restricted diets in management of motor fluctuations.

## Clinical effects of protein-restricted diets

### Low-protein diet (LPD)

Some early studies have presented that a diet containing ~0.5–0.8g protein per kilogram of body weight per day helped to improve motor fluctuations and lower disability scores in parkinsonian patients (Table 1; Mena and Cotzias, 1975; Juncos et al., 1987; Carter et al., 1989). Moreover, Juncos et al., have advocated the use of a low-protein diet (LPD) that adheres to recommended dietary allowance (RDA) protein guidelines (0.8 g protein/kg /day) in PD patients. In the study by Juncos *et al*. on 6 fluctuators with stage III/V PD, while all the subjects experienced abrupt emergence of parkinsonian symptoms in 30–60 minutes after a protein intake in excess of RDA, a diet meeting RDA resulted in no abrupt loss of response to levodopa (Juncos et al., 1987). Besides, Juncos *et al*. also demonstrated that dividing the protein intake into more frequent but smaller meals is not necessary in a LPD, as long as the total daily intake of protein was fixed (Juncos et al., 1987). In order to rule out placebo effects, two double-blinded researches were performed later on (Tsui et al., 1989; Croxson et al., 1991). By adding LNAAs or placebo to LPDs in a crossover design, Yen *et al*. found 3/8 patients with fluctuating parkinsonism had markedly improved motor function, but no overall significant difference was observed (Croxson et al., 1991). In the study by Tsui et al., 10 PD patients were given high- and LPDs, which tasted and looked alike, each for 1 week. On LPDs, the patients exhibited significantly better motor performance that did not correlate with plasma concentrations of levodopa, indicating LPD can enhance the effect of levodopa at a central level (Tsui et al., 1989). Although a small number of patients were included, these double-blinded trials have furtherly confirmed the effect of LPD in attenuating motor fluctuations.

**Table 1:** Studies on the clinical effects of Protein-restricted diets among PD patients with motor fluctuations.

**Protein-restricted diet**	**Trial duration**	**Number of subjects**	**Characteristics of subjects**	**Duration of PD (range)**	**Daily protein intake (trial)**	**Daily protein intake (control)**	**Effects on motor function**	**Rate of responsiveness**	**Drug dosage**	**Reference**
LPD	5 days	5	Idiopathic PD with marked fluctuations	19.4 yrs (14–25)	0.8 g/kg	1.6 g/kg	“on” time: ↑16% of the waking day; AIMS score: ↑1.9 points	–	–	Carter et al., 1989
	7 days	6	Idiopathic PD with fluctuations; Hoehn-Yahr stage III–V	14 yrs (8–19)	0.78 g/kg (divided into 3 or 6 meals, *n* = 6)	1.2 g/kg (*n* = 3)	Elimination of “off” state after the protein load (no details reported)	–	–	Juncos et al., 1987
	7 days	8	Idiopathic PD with or without fluctuations	–	0.5 g/kg	1 g/kg	Mean disability score at 3pm: ↓3.4 points (maximal disability = 100; normality status = 0)	–	–	Mena and Cotzias, 1975
PRD	1 day	8	Idiopathic PD with unpredictable fluctuations	12 yrs (5–20)	7 g before dinner	160 g before dinner	“Immediate clinical benefit”	86%	↓538 mg/day (39%, *n* = 6)	Pincus and Barry, 1987b
		7	Idiopathic PD with non-response to LD/CD	8 yrs (3–12)			Northwestern Disability Score: ↓17 points	86%	↓11 and 14% respectively (*n = 2*)	
	5 days	7	PD with marked and unpredictable fluctuations	16 yrs (8–22)	7 g before dinner	160 g before dinner	A shift from bradykinesia to dyskinesia	100%	↓28% (*n* = 5)	Pincus and Barry, 1987c
	5 days	5	Idiopathic PD with marked fluctuations	19.4 (14–25)	0.8 g/kg (7 g before dinner)	1.6 g/kg	“on” time: ↑26% of the waking day; AIMS score: ↑2.5 points	–	–	Carter et al., 1989
	≥4 weeks	30	PD with disabling fluctuations	14.7 yrs	7 g before dinner	–	Mean “off” time: ↓3.5 h/d (*n* = 14); “Improved peak motor performance” (no details reported, *n* = 8); UPDRS scores: no significant change	60.7%	↓ (no details reported, n = 5)	Riley and Lang, 1988
		8	PD with inadequate response to LD	4.1 yrs			No significant benefit	0%	–	
	8 weeks	11	PD with unpredictable fluctuations	–	10 g before dinner	–	Improved daytime mobility (no details reported, *n* = 7)	63.6%	–	Pare et al., 1992
	1–12 months (mean, 5 months)	11	Idiopathic PD with disabling daily fluctuations	12 yrs (6–20)	7 g before dinner	160 g before dinner	Northwestern Disability Score: ↓24 points; AIMS score: ↑14 points	82%	↓41% (*n* = 8)	Pincus and Barry, 1987a
	1–12 months	16	Idiopathic PD with daily fluctuations; Hoehn-Yahr stage II–IV	9 yrs (3–14)	0.8 g /kg (“virtually free of protein” before dinner)	–	NYU Score: ↓11 points (Bradykinesia: ↓11; Rigidity: ↓8; Tremor: ↓7; Gait: ↓14; the values are expressed in % of NYU Score)	100%	–	Bracco et al., 1991
	2–12 months (mean, 6 months)	7	Idiopathic PD; 5 with moderate fluctuations; 2 with marked fluctuations	–	0.5 g/kg (3g at breakfast, 3g at lunch and the rest at dinner)	–	Improved stability of disability scores (no details reported, *n* = 6)	100%	↓20% and 35% respectively (*n* = 2)	Mena and Cotzias, 1975
	1 week–16 months (mean, 7 months)	16	PD with drug-resistant “off” periods but without dyskinesia	8 yrs (3–15)	7 g before dinner	160 g before dinner	NYU Score: ↓22 points	88%	↓6 mg/day	Pincus and Barry, 1988
	9 months	277	Idiopathic PD; Mean Hoehn-Yahr stage: 2.4; MMSE > 24 points	8.8 yrs	1.1 g/kg	–	“off” score by UPDRS-Part IV: 1.1 points (↓0.4 points)	–	488 mg/day (↓68 mg/day)	Barichella et al., 2016a
		233 (control)	Idiopathic PD; Mean Hoehn-Yahr stage: 2.5; MMSE > 24 points	9.6 yrs	–	1.3 g/kg	“off” score by UPDRS-Part IV: 1.5 points	–	556 mg/day	
	2 days–3 weeks	43	PD with unpredictable fluctuations	13.7 yrs (4–32)	10 g before dinner	–	Worst NYU Score: ↓12.7 points; “on” time: ↑59% of observation hours; Best walking time: ↓9.7s	100%	↓54.5 mg/day	Karstaedt and Pincus, 1992
	14–48 months (mean, 33.6 months)	30		–		–	Worst NYU Score: ↓13.2 points	69.8%	↓72.1 mg/day	
PRD with use of LPP	12 weeks (6 weeks for each diet)	6	PD with fluctuations (“off” state for at least 30 mins within 5 h after lunch)	21 yrs (11–27)	0.8–1 g/kg ideal body weight (87.3% at dinner, with use of LPP)	0.8–1 g/kg ideal body weight (64.1% at dinner, without use of LPP)	Dyskinetic “on” time: ↑1.5 h/d; “off” time: ↓1.5 h/d	100%	–	Barichella et al., 2007
	2 months	18	PD with fluctuations (“off” state for at least 30 mins within 5 h after lunch); Hoehn-Yahr stage II–III	11.5 yrs	0.8 g/kg ideal body weight (85.3% at dinner)	0.8 g/kg ideal body weight (39.8% at dinner)	“on” time: ↑1.9 h/d; “off” time: ↓1.8 h/d	50%	↓45.8 mg/day (9.2%, *n* = 6)	Barichella et al., 2006

*PD, Parkinson's disease; LPD, low-protein diet; PRD, protein-redistribution diet; LPP, low-protein Products; LD, levodopa; CD, carbidopa; MMSE, Mini-Mental State Examination; AIMS, Abnormal Involuntary Movement Scale; NYU Score, New York University Disability Score; UPDRS, Unified Parkinson Disease Rating Scale*.

As a dietary strategy to improve motor fluctuations in PD, LPD is not only effective, but also simple to understand and follow. Nevertheless, it is noteworthy the protein intake of 0.8 g/kg/day recommended by current RDA may not be adequate for the elderly to maintain physical function and optimal health (Wolfe et al., 2008; Volpi et al., 2013). More specifically, a protein intake of 1.5 g/kg/day was suggested to be a more reasonable level in older adults (Wolfe et al., 2008). On one hand, it requires further investigation on the long-term benefit of the LPD adhering to current RDA. On the other hand, a revised LPD that raises the daily protein intake to 1.5 g/kg/day is worth testing for efficacy.

In addition, the LPD is also found to alleviate motor dysfunction in parkinsonian mice (Yang and Cheng, 2010; Wiesner et al., 2015). This effect may be related to activation of autophagy pathway, reduced activation of microglia, lower levels of proinflammatory cytokines and less loss of tyrosine hydroxylase (TH)-positive neurons (Yang and Cheng, 2010; Wiesner et al., 2015). These studies provide evidence for the protective effect of LPD om motor function at the cellular and molecular levels. However, the parkinsonian mice in the studies were not animal models for motor fluctuations, and were not on levodopa therapy either. Thus, the analysis of these studies is beyond the scope of this review.

### Protein-redistribution diet (PRD)

In order to maximize daytime motor function and improve QOL of the patients to a greater extent, a protein-redistribution diet (PRD) has also been applied in PD patients with fluctuating response to levodopa (Pincus and Barry, 1987a,b,c, 1988; Riley and Lang, 1988; Carter et al., 1989; Bracco et al., 1991; Karstaedt and Pincus, 1992; Pare et al., 1992). Due to the marked interaction between dietary proteins and the response to levodopa, protein intakes at breakfast and lunch are likely to be a significant contributor to diurnal motor fluctuations. Meanwhile, diurnal motor fluctuations are considered to limit QOL of the patients more greatly than the fluctuations at night. Therefore, the protein intake in a PRD is limited to 7 g before the evening meal, but is unrestricted afterwards until bedtime. More specifically, patients on a PRD consume low-protein food including cereal products (e.g., bread, rice, and pasta), fruit, and vegetables, for breakfast and lunch, and are only allowed to have high-protein food including dairy products, eggs, legumes, fish, and meat for dinner. Around half of the studies also limited the total protein intake to 0.8 g/kg/day.

Previous studies have exhibited that PRD can markedly ameliorate motor fluctuations in patients with various stages of PD (Table 1; Pincus and Barry, 1987a,b,c, 1988; Riley and Lang, 1988; Carter et al., 1989; Karstaedt and Pincus, 1992; Pare et al., 1992). Most subjects in studies of PRD experienced an improved response to levodopa during the diet, with a percentage from 60.7 to 100%. The clinical benefits from PRD included elimination of bradykinesia emerging on a high-protein diet, elevation in “on” time, reduction in “off” time and better peak motor performance (Table 1). The disability scores assessed by different methods, including New York University (NYU) Rating Scale and Northwestern Parkinsonian Disability Scale, were consistently improved on PRD. Additionally, about two-thirds of the studies on PRD from 1973 to 2009 reported above 80% of their subjects responded well to a PRD (Cereda et al., 2010a), suggesting PRD is effective to enhance motor performance in a large proportion of fluctuating parkinsonians. This result also implies the prevalence of PIL may be underestimated in the above-mentioned study of Virmani et al., where PIL was reported to occur in 12.4% of fluctuators (Virmani et al., 2016).

Carter et al. compared the effectiveness of LPD and PRD in 5 parkinsonians with marked fluctuations, and found PRD led to further amelioration of motor fluctuations than LPD did (Table 1). However, there is only one study trying to compare the efficacy of these two protein-restricted diets, and the sample of this study is quite small. Therefore, more studies are still needed to confirm a higher effectiveness of PRD in improving fluctuations.

Beside the fluctuating motor function, PRD also influences the dosage of anti-parkinsonian drugs (Pincus and Barry, 1987a,b,c; Karstaedt and Pincus, 1992). In a study published by Pincus et al. PRD decreased daily levodopa dose from a mean of 1243 to 907 mg in 7 patients with fluctuations (Pincus and Barry, 1987c). In another study on 11 subjects with disabling fluctuations, PRD permitted 8 patients to reduce daily levodopa dose by a mean of 41% (Pincus and Barry, 1987a). Consistently, a positive correlation between daily protein intake and levodopa dosage was observed in the above-mentioned study on 45 Italian PD patients (Marczewska et al., 2006). In addition to a reduction in levodopa requirement, there were 38–100% of the subjects discontinuing all previously-used adjuvant therapy on a PRD (Pincus and Barry, 1987a,b,c). Such reduced requirements for levodopa or other adjuvant drugs indirectly prove the enhanced clinical efficacy of levodopa on a PRD. Furthermore, a reduced drug dosage during the diet may help to attenuate adverse effects of the anti-parkinsonian drugs, like dyskinesia, nausea, and hallucinations (Connolly and Lang, 2014). However, it has been reported that some patients experienced more significant dyskinesia and psychosis upon introduction of PRD, due to a better response to levodopa (Pincus and Barry, 1987a,b, 1988; Riley and Lang, 1988; Carter et al., 1989; Barichella et al., 2006). A subsequent dose modulation may probably reverse the precipitated levodopa side effects, but its effectiveness has not been clearly stated in any previous study. Therefore, further studies are still warranted in order to better understand the impact of PRD on the side effects of dopaminergic treatment.

Most studies on PRD were performed back in the 1980s and the early 1990s, and have been limited by small numbers of patients. Nevertheless, Barichella et al. recently performed a large observational study in 510 parkinsonians on levodopa therapy, and compared the motor function and levodopa dosage of 277 patients who adhered to PRD and 233 patients who did not. This study revealed that the patients following PRD indeed experienced a significantly improved “off” score by Unified Parkinson Disease Rating Scale (UPDRS)-Part IV as well as a markedly decreased levodopa dosage (Barichella et al., 2016a). The inverse relation between PRD and motor fluctuaions was furtherly confirmed via multivariate regression analysis including gender, age of onset, duration of PD and dosage of levodopa. (Barichella et al., 2016a). The study also found that an excess of protein intake by 10 g over RDA was corresponded to an increase in levodopa dosage by 0.7 mg/kg/day, confirming the negative correlation between protein intake and therapeuatic efficiency of levodopa (Barichella et al., 2016a). Regrettably, the major limitation of this study is a cross-sectional design which lacks information about the duration of PRD in 277 adherents. Additional research, especially cohort studies, will be required in order to explore the cinical benefits of a prolonged PRD.

Although, the rate of responsiveness to PRD is considerably high in fluctuators, the response to the diet still varies from patient to patient. While some patients acquired a constant clinical benefit from PRD, others showed a transitory or mild response to the diet, or even experienced worse parkinsonism symptoms and fluctuations (Bracco et al., 1991; Karstaedt and Pincus, 1992). Only a few studies have identified the characteristics of patients who showed a better response to PRD, and the results are mixed. A study on 16 fluctuators found that patients' response to PRD correlated inversely with their duration of fluctuations, but did not significantly correlate with disease duration, age at onset, duration of levodopa treatment, or levodopa dosage (Bracco et al., 1991). Nevertheless, Giménez-Roldán et al. found that those with unsatisfactory response to PRD were also characterized by a younger age at onset beside longer duration of fluctuations. Furthermore, the study suggested that the patients showing fluctuations in the later stages of the disease, especially those with unpredictable “on-off” effects and dose-failures unrelated to meals, were more likely to respond poorly to PRD (Gimenez-Roldan and Mateo, 1991). In a study by Riley et al. the patients with no response to PRD showed a shorter duration of PD as well as a shorter duration of levodopa therapy (Riley and Lang, 1988). As mentioned previously, the onset of PIL observed in the study by Virmani et al. implies it will take the patients almost 13 years after the onset of PD or 8 years after the initiation of levodopa, to develop PIL and become responsive to a protein-restricted diet (Virmani et al., 2016). Together, the previous studies indicate the responsiveness to PRD in fluctuating parkinsonians is positively correlated with duration of PD and duration of levodopa therapy, and is negatively correlated with duration of motor fluctuations.

It also remains controversy whether the patients with inadequate response to levodopa therapy will show improved motor function during a PRD or not. Riley et al. reported none of the “levodopa-resistant” patients, who had shown unsatisfactory response to levodopa since its introduction, benefited significantly from a PRD in their study. This finding indicated although PRD might be able to enhance the response to levodopa in PD patients, its ability to initiate a response to the drug was limited (Riley and Lang, 1988). In contrary, a study conducted by Pincus et al. found that PRD led to immediate sensitivity to carbidopa-levodopa (Sinemet) in 86% of the “non-responders” and reduced their mean disability score by 17 points. Additionally, there was one non-responder starting to take CD/LD which had been ineffective in him/her before receiving a PRD (Pincus and Barry, 1987b). However, the study did not clarify whether the so-called “non-responders” had never benefited from CD/LD, or had shown a response to the drug initially but lost it with prolonged treatment. The body of evidence available is insufficient to prove PRD's ability to initiate a response to levodopa therapy, and additional investigation is needed.

Despite the significant clinical benefits of PRD, there are concerns about the acceptability of the diet in PD patients. According to a systematic review of Cereda et al., acceptability of PRD was considerably high upon introduction, whereas it seemed to progressively decrease over time. Levodopa side effects such as hallucinations, confusion, and depression could be precipitated by a better response to the drug on PRD, and were the main contributors to drop-outs at the beginning. Nevertheless, the acceptability of a prolonged PRD was affected more by changes in dietary habits (Pincus and Barry, 1988; Riley and Lang, 1988; Cereda et al., 2010a). Due to a great difference between PRD and common dietary habits, some patients complaint it was subjectively difficult to get accustomed to PRD (Riley and Lang, 1988), which is also referred as the “logistic difficulties” of the diet (Riley and Lang, 1988; Barichella et al., 2009; Cereda et al., 2010a,b). A study by Paré et al. showed that one third of the subjects (4 of 11) disliked the diet, complaining about the hunger before supper and the lack of variety in food, particularly at lunch (Pare et al., 1992).

Compared with LPD, the use of PRD in parkinsonians has been investigated more thoroughly by the previous studies. The effectiveness of PRD in ameliorating motor fluctuations appears to be guaranteed. However, the potential side effects of the diet, including dyskinesia, worse nocturnal motor symptoms, and changes in nutritional status of the patients, have not been profoundly discussed. On the other hand, although PRD is effective in patients with stage I–IV PD, and appears to be more effective than LPD, the low acceptability may be a problem for prolonged adherence to the diet. Thus, it is more reasonable to use PRD in the advanced patients who cannot obtain satisfactory benefit from LPD. Otherwise, for the patients earlier in the disease course, LPD is an easier way to improve motor fluctuations and is worth trying first.

### Use of low-protein products (LPP)

The special Low-protein Products (LPP) originally designed for renal failure patients have been used in PRD with the aim to improve the acceptability of the diet (Pare et al., 1992; Barichella et al., 2006, 2007). Like the other protein-restricted diets, a PRD with use of LPP can also ameliorate fluctuations in PD patients (Table 1). Compared with an ordinary diet, a 2-month LPP diet in 18 patients led to reductions in both total and postprandial “off” time by 107 and 30 min, respectively. The total and optimal postprandial “on” time also, respectively, extended by 114 and 30 min (Barichella et al., 2006). In another study on 6 fluctuating parkinsonians, a PRD with use of LPP also significantly shortened 24-h “off” time and increased 24-h dyskinetic “on” time of the patients (Barichella et al., 2007).

Although, these results indicate a PRD involving consumption of LPP helps to ameliorate motor fluctuations in parkinsonians, it is still unclear whether these commercial prepared products can enhance the patients' acceptability to PRD. The high cost, poor palatability and limited availability are probably the major obstacles hindering wider acceptance of these products (Pare et al., 1992). Still, LPP is a reasonable option for the patients who try to gain a wider selection of food on PRD.

### Side effects of protein-restricted diets on motor function

As a result of a better response to levodopa, the protein-restricted diets not only ameliorate motor fluctuations of the patients, but also induce several motor side effects. Dyskinesias, or choreic movements, are the principal side effect of protein-restricted diets on motor function (Pincus and Barry, 1987a,b; Riley and Lang, 1988; Carter et al., 1989; Barichella et al., 2006). Pincus et al. indicated 82% of their subjects experienced a marked relief of parkinsonian symptoms on a PRD, but all these patients demonstrated dyskinesia at the same time. The Abnormal Involuntary Movement Scale (AIMS) score of the subjects rose by 14 points during PRD with a mean duration of 5 months (Pincus and Barry, 1987a). Likewise, Carter et al. reported the mean AIMS dyskinesia score of 5 patients with marked fluctuations increased by 1.9 points during a LPD (0.9 vs. 2.8 points), and increased by 2.5 points during a PRD (0.9 vs. 3.4 points; Carter et al., 1989). By comparing the observational data of 277 PRD adherents and 233 controls, Barichella et al. found that the patients receiving a PRD got a higher mean dyskinesia score by UPDRS-Part IV compared with those who did not follow the diet, but the difference was not statistically significant (Pincus and Barry, 1987c; Barichella et al., 2016a). The development of dyskinesia also exists in the patients who use LPP during a PRD. As mentioned above, the daily dyskinetic “on” time can extend significantly during an LPP diet. The patient global improvement (PGI) questionnaire, however, indicated the patients on the diet still experienced a clinical benefit in spite of increased dyskinesia (Barichella et al., 2007). Anyway, additional research is required to explore the severity and incidence of dyskinesia induced by protein-restricted diets, as well as its influence on disability levels of the patients.

A modulation of levodopa dosage is the major method to control dyskinesia on a protein-restricted diet. In two studies of Pincus et al., respectively, 75 and 73% of the subjects required a reduction in levodopa dosage to avoid chorea (Pincus and Barry, 1987a,b). In addition, there were two outpatients who noticed that a reduced dosage of Sinemet helped to improve their dyskinesia without shortening their “on” time (Carter et al., 1989). Beside a smaller medication dosage, a pro re nata (PRN) protein supplement was also reported to reverse dyskinesia on PRD by two patients (Riley and Lang, 1988). Compared with a low protein-high carbohydrate diet, Berry et al. indicated that a diet with a carbohydrate: protein ratio of 5:1 was less likely to cause dyskinesia as well as fluctuations in levodopa concentrations (Berry et al., 1991). However, the effectiveness of these approaches to relieve dyskinesia on protein-restricted diets has only been reported in small numbers of patients (*n* ≤ 11) without any statistical analysis. Additional large-sample studies or double-blinded trails are still required.

Greater severity in nocturnal motor symptoms may also be problematic in PRD. PD-related motor symptoms, including nocturnal akinesia, dystonia, painful cramps, tremor, and difficulty turning in bed, are common sleep disorders in patients with motor fluctuations (Stocchi et al., 1998; Barone et al., 2004). Moreover, nocturnal akinesia was found to significantly lower QOL and affect emotional well-being in PD patients (Chapuis et al., 2005). As the majority of dietary protein in PRD is consumed at dinner, the diet may worsen the above-mentioned sleep disorders and reduced QOL in adherents. Barichella et al. reported there was no significant difference in sleeping hours between the patients on LPD and PRD, which implies the heavy protein load during the evening in a PRD does not impair the motor function and QOL of the patients (Barichella et al., 2006). By contrast, Carter et al. found the percentage of mobile hours after dinner, which was 63% on LPD, declined to 52% on PRD (Carter et al., 1989). However, the study did not show the changes in sleep hours or nocturnal motor symptoms of PRD adherents. To our best knowledge, none of the existing studies have provided direct evidence regarding the influences of PRD on nocturnal motor function or overall QOL of the adherents. Therefore, further investigation is still needed.

### Side effects of protein-restricted diets on nutritional status

PD patients are likely to show lower body mass index (BMI) than healthy individuals (van der Marck et al., 2012), and their BMI is inversely associated with disease severity (van der Marck et al., 2012; Barichella et al., 2016a; Wills et al., 2016). Thereby, weight loss is another major concern regarding side effects of PRD. Nevertheless, mild weight loss is a common problem during a PRD no matter whether LPP is consumed or not (Pincus and Barry, 1987c; Riley and Lang, 1988; Pare et al., 1992; Barichella et al., 2006, 2007; Cereda et al., 2010c). In a follow-up of more than 4 weeks, a mean body weight loss of 1.8% was observed in 30 PRD adherents (Riley and Lang, 1988). Similarly, in a recent study, 18 fluctuating patients experienced a reduction in body weight by a mean of 0.32 kg during a 2-month PRD (Riley and Lang, 1988; Barichella et al., 2006).

The weight loss in PRD may be related with less diurnal motor fluctuations during the diet. On a PRD, longer “on” time and shorter “off” time are likely to increase the physical activity and metabolic rate of the patients, and thus result in an elevation in their daily energy expenditure (Barichella et al., 2006, 2007; Cereda et al., 2010c). In a 8-week follow-up, the mean body weight of 11 subjects decreased by 1.11% within the first two weeks of PRD but remained stable in the following 4 weeks, which indicates the weight loss is probably a result of the changes in dietary habits (Pare et al., 1992). Although, most of the studies pointed out the weight loss during PRD, if any, was slight and the patients could maintain their weights above ideal levels, Karstaedt et al. reported that two subjects in their trial discontinued PRD because they were unable to keep an ideal body weight during the diet (Karstaedt and Pincus, 1992).

In contrast to the above findings, there also is evidence showing PRD is associated with an increased body weight. In the large cross-sectional study by Barichella et al. the patients on PRD had higher BMI in spite of lower protein and calorie intakes (Barichella et al., 2016a). As the weight loss during a PRD is related with changes in dietary habits upon introduction of the diet, it is reasonable that adherence to PRD for an extended period of time lasting several years may eventually result in a stable body weight. Regrettably, the duration of PRD in this study was not stated. Moreover, the increased body weight can also be explained by the potentially lower energy expenditure during a PRD which results from a reduced levodopa dosage and alleviated levodopa-related dyskinesia on the diet. In a case report published by Barichella et al. a patient who used to experience a dramatic weight loss induced by dyskinesia, showed a satisfactory increase in body weight through a combination therapy of a protein-restricted diet and high-calorie oral supplements, demonstrating the positive effect of protein-restricted diets on weight gain in PD patients (Barichella et al., 2011). However, the report did not clarify which protein-restricted diet was used in this case.

As for LPD, the body weight levels of adherents have not been analyzed in any previous study. Since both the diets limit the daily protein intake to 0.8 g/kg, LPD is likely to have a similar effect on body weight as PRD. From the studies mentioned above, the risk of weight loss in patients following PRD or LPD is still unclear. Although, further investigation is needed, it is recommended all the patients on a long-term protein-restricted diet to increase daily energy intake properly and be monitored for weight loss.

The majority of the studies have reported there were no visible signs of nutrient deficiency in a protein -restricted diet during follow-ups ranging from 6 weeks to 10 months (Mena and Cotzias, 1975; Pincus and Barry, 1987a,b; Riley and Lang, 1988; Barichella et al., 2006). Paré et al. monitored the levels of 14 major nutrients in PRD adherents during the 8-week follow-up mentioned above. It was revealed that the intake of protein, calcium, iron, and potassium were all decreased significantly during a PRD, but only the intake of calcium was below a recommended nutrient intake, suggesting the necessity of calcium supplement during a long-term PRD (Pare et al., 1992). Riley et al. found only 1 in 33 patients experienced a fell in serum albumin during at least 4 weeks of follow-up (Riley and Lang, 1988). Moreover, Mena et al. reported there were no significant changes in albumin-globulin ratio of all 8 patients during a short-term LPD. Similarly, a long-term (2 months to 1 year) RPD did not result in drops in albumin-globulin ratios or serum protein levels either (Mena and Cotzias, 1975). The recent study performed by Barichella et al. which has been mentioned previously, helps to provide a more complete picture of nutritional status in PRD adherents. The patients following PRD were found to consume less protein, as well less micronutrients (like zinc, calcium, and Vietnam B12) whose main sources were high-protein food of animal origin. However, all the nutrient intakes seemed to meet daily requirements (Barichella et al., 2016a). Taken together, these studies suggest that the risk of nutrition deficiency during a long-term PRD is low, whereas ongoing nutrient monitoring is still needed.

Due to an association between PD and a redistribution in body composition from muscle to fat (Barichella et al., 2016b; Lindskov et al., 2016), the effects of protein-restricted diets on patients' muscle mass and nitrogen balance are also worth noting. A cross-sectional study conducted by Maryanne et al. showed that a group of 17 parkinsonians, whose daily protein intake was ~1.1 g/kg, experienced a negative nitrogen balance of −1.8 g/day, implying the protein intake in LPD or PRD may not be sufficient to maintain neutral nitrogen balance in PD patients (Zilli Canedo Silva et al., 2015). Moreover, as mentioned above, although the daily protein intake in both LPD and PRD meet the current RDA standard (0.8 g protein/kg/day), a higher intake level of 1.5 g protein/kg/day may be more reasonable for the elderly (Wolfe et al., 2008; Volpi et al., 2013). Therefore, further studies are needed to explore the changes in body composition and nitrogen balance of the patients receiving a PRD or LPD. Additionally, the association between patients' muscle mass and their response to levodopa also required further clarification. Before firmer conclusion can be drawn, special attention should be paid to patients' muscle mass and nitrogen balance during a protein-restricted diet.

## Conclusions

Levodopa competes with LNAAs for the same saturable LNAA system in intestinal mucosa and at blood brain barrier, resulting in variability in levodopa pharmacokinetics related to dietary proteins. Thereby, protein loading raises plasma LNAA levels, reduces and retards the brain uptake of levodopa, and diminishes the response to the drug in fluctuating parkinsonians. Such protein interaction with levodopa appears about 13 years after the onset of motor symptoms or 8 years after the initiation of levodopa therapy, and correlates with higher severity of motor fluctuations.

Protein-restricted diets, including LPD, PRD, and PRD with use of LPP, are all effective in ameliorating motor fluctuations. Among these diets, PRD has been investigated most thoroughly, and has been confirmed to be effective in improving motor response and reducing levodopa dosage in fluctuating patients with various stages of PD. Almost 60.7 to 100% of the fluctuators on levodopa respond well to PRD, and the responsiveness is positively correlated with duration of PD and duration of levodopa therapy. The major problem for prolonged adherence to PRD is low acceptability. Although, LPP offers PRD adherents a wider selection of food, it is still not a promising way to enhance the patients' acceptability to the diet.

Dyskinesias are the major side effect of protein-restricted diets on motor function. The methods to control dyskinesia on the diets include a modulation of levodopa dosage, a PRN protein supplement, and a diet that balances carbohydrate and protein, whose effectiveness all requires further confirmation in future studies. In PRD adherents, greater severity in nocturnal motor symptoms may also be problematic. The influences of PRD on nocturnal motor function or overall QOL still need to be clarified.

The risk of nutrition deficiency during a long-term protein-restricted diet is low, but the adherents' muscle mass and nitrogen balance still require special attention. Another major concern regarding the nutritional status during the diets is weight loss. Evidence on this issue is mixed, indicating weight loss and weight gain may both be encountered in patients following PRD and LPD.

In light of these conclusions, the authors make several recommendations concerning protein-restricted diets. Due to the interactions between dietary proteins and levodopa pharmacokinetics, the patients on levodopa therapy are recommended to take the drug on an empty stomach. In the patients with motor fluctuations, protein-restricted diets including LPD and PRD are both effective and economical approaches to improve fluctuating response to levodopa. In patients at an earlier stage of PD, LPD can be tried first. As the disease progresses and motor fluctuations become more severe, PRD, which is likely to be more efficacious in improving fluctuations, is an alternative diet for the patients with unsatisfactory response to LPD. Moreover, LPP is a reasonable option for the patients who try to obtain a wider selection of food on PRD, but is not necessary in all patients following the diet. No matter which protein-restricted diet is followed, all patients require monitoring for dyskinesia, weight loss, as well as signs of malnutrition.

## Author contributions

Conception, LW, NX, JH, SG, LL, CH, GZ, HJ, KM, YX, XX, JL, JYL, and TW; Collection of materials, LW, NX, JH, SG, LL, CH, and GZ; Writing of the first draft, LW, NX, JH, HJ, KM, YX, XX, JL, and JYL; Review and critique, NX, JH, and TW; All authors read, revised, and approved the final manuscript.

### Conflict of interest statement

The authors declare that the research was conducted in the absence of any commercial or financial relationships that could be construed as a potential conflict of interest.

## Abbreviations

PD: Parkinson's Disease
LNAA: large neutral amino acids
LPD: low-protein diet
PRD: protein-redistribution diet
MAO-B: monoamine oxidase type B
COMT: catechol-O-methyltransferase
QOL: quality of life
IR: immediate-release
CD: carbidopa
LD: levodopa
AUC: concentration time curve
AADC: amino acid decarboxylase
BBB: blood brain barrier
PIL: protein interaction with levodopa
NYU: New York University Rating Scale
RDA: recommended daily allowance
UPDRS: Unified Parkinson Disease Rating Scale
LPP: low-protein products
AIMS: Abnormal Involuntary Movement Scale
BMI: body mass index.
